# Epidemiological, clinical and climatic characteristics of dengue fever in Kaohsiung City, Taiwan with implication for prevention and control

**DOI:** 10.1371/journal.pone.0190637

**Published:** 2018-01-02

**Authors:** Chiu-Jung Chang, Colin S. Chen, Chien-Jung Tien, Mei-Rou Lu

**Affiliations:** 1 Kaohsiung Municipal Kai-Syuan Psychiatric Hospital, Kaohsiung, Taiwan; 2 Department of Biotechnology, National Kaohsiung Normal University, Kaohsiung, Taiwan; Institut Pasteur of Shanghai Chinese Academy of Sciences, CHINA

## Abstract

**Background:**

The early identification of dengue infection is essential for timely and effective quarantine and vector control measures for preventing outbreaks of the disease. Kaohsiung City is responsible for most of the dengue cases in Taiwan. Thus, this study aims to identify major factors involved in the prevalence of dengue fever by analyzing the epidemiological and clinical characteristics, and to establish associations between weather parameters and dengue occurrence in this City.

**Methods:**

A retrospective study was conducted with 3,322 confirmed dengue cases. Appropriate statistical methods were used to compare differences and correlations between dengue occurrence and demographic, clinical and weather parameters.

**Results:**

The outbreak of dengue fever was found to be initiated by imported cases of dengue viruses from other endemic countries. Most of the confirmed cases were not reported to the health authority during the first visit to a doctor, and it took a median of 5 days after the appearance of the first syndromes for medical personnel to report suspected dengue cases. Accordingly, *Aedes* mosquitoes would have enough time to be infected and transmit the dengue virus. The diagnosis and notification criteria should not only include common symptoms of fever, myalgia, headache, skin rash and arthralgia, but should also be adjusted to include the most frequent symptoms of loss of appetite and feeling thirsty to shorten the notification time. Significantly positive correlations were found between the number of confirmed cases and weather parameters (*i*.*e*., temperature, rainfall and relative humidity) at a time lag of 1 month and 2 months. The predictive models for dengue occurrence using these three parameters at a 2-month lag time were established.

**Conclusions:**

The surveillance of imported cases, adjustment of notification criteria and application of climatic predictive models would be helpful in strengthening the dengue early warning surveillance system.

## Introduction

The global distribution of dengue fever (DF) in tropical and subtropical regions has caused a serious public health problem. According to the World Health Organization (WHO), an estimated 50–100 million dengue infections occur each year, and close to 75% of the population exposed to DF are in the Asia-Pacific region [1]. There is no exception for Taiwan, which is located in southeastern Asia. The occurrence of DF was first recorded in Taiwan in 1779. The most severe outbreaks of DF in Taiwan occurred in 1915, 1931, 1942, 1988 and 2002. This disease became endemic, with yearly and frequent outbreaks thereafter. Most of the dengue cases occurred in southern Taiwan, particularly in Kaohsiung [2]. Thus, monitoring trends in the distribution and spread of DF over time in Kaohsiung would assist in the rapid detection of epidemics for early intervention.

DF is ranked as the most important mosquito-borne viral disease in the world [1]. Dengue virus (DV), the pathogen of DF, has 4 distinct serotypes (DENV-1~4) of the genus *Flavivirus* [3]. Patients infected by DV have a wide range of non-specific clinical manifestations, including high fever, pain in 5 characteristic areas (severe frontal headache, retro-orbital pain, bone pain, myalgia, and arthralgia), and maculopapular rash [3]. Some diseases, such as influenza, measles, enteric fever, leptospirosis, typhus fever, malaria, etc., have similar manifestations. Distinguishing DF from other dengue-like diseases is important for early diagnosis and treatment. In addition, several studies had shown that the clinical spectrum of DF varied among different regions [4,5]. Thus, understanding patterns of the clinical spectrum of DF in Kaohsiung, Taiwan would aid the early recognition of this disease. Although there is no specific treatment for DF, early detection and access to proper medical care has been found to lower fatality rates to below 1% [6].

DF has been categorized as a Class II notified infectious disease in Taiwan, and it is controlled by the National Infectious Diseases Notification Surveillance System (NIDNSS) at the Centers for Disease Control, Taiwan (TCDC). Once suspected cases are found, notification of NIDNSS should be made within 24 hours. The local primary health center then initiates public health measures (e.g., an outbreak investigation, residual surface treatments of insecticides to reduce the density of vectors, and the elimination of containers for oviposition and the development of the aquatic stages) after cases are confirmed as dengue infection by the National Virus Diagnosis Laboratory in TCDC. Therefore, early notification of NIDNSS would decrease the incidence of infection and prevent outbreaks of the disease; however, most suspected cases were reported to the health authority after visiting doctors 2–3 times [7]. It took approximately 4 d after the appearance of the first syndromes for doctors to report suspected cases due to a lack of awareness about DF by doctors, nurses and medical staff [8]. However, infected humans in the febrile phase can transmit DV (for 4–5 d; maximum 12 d) via female *Aedes aegypti* and *Ae*. *albopictus*, principally *Ae*. *aegypti* after their first symptoms appear. Once DV has been ingested by *Aedes* mosquitoes for 4–10 d, an infected mosquito is capable of transmitting the virus for the rest of its life [6]. Thus, prevention of infected human-vector contact and initiation of vector control measures after the notification of suspected cases was too late to achieve a significant impact. In addition, the biology, abundance and distribution of *Aedes* mosquitoes have been demonstrated to be directly affected by changes in weather parameters (*e*.*g*., temperature, rainfall and relative humidity) [9–11]. Therefore, the objectives of this study were to analyze the epidemiological and clinical characteristics of DF and to establish associations between weather parameters and the occurrence of dengue cases in Kaohsiung City, Taiwan in order to improve the localized dengue surveillance and early warning system.

## Materials and methods

### Study site

Kaohsiung City is by area the largest special municipality (approximately 2947.6 km^2^) and is the second most populous (approximately 2.77 million people) in Taiwan since December 25, 2010 when Kaohsiung City merged with Kaohsiung County to form a special municipality. It has a tropical monsoon climate, with an average annual temperature and rainfall of 25.1°C and 2,549.4 mm, respectively. This climate is suitable for the development of *Aedes* mosquitoes.

### Case definition and notification of dengue infection

According to the regulation of TCDC, patients with fever (≥ 38°C) and two or more of the manifestations, headache, retro-orbital pain, myalgia, arthralgia, rash, hemorrhagic manifestations and leukopenia should be reported to NIDSS as suspected cases. The suspected case was confirmed using laboratory diagnosis. The one-step real-time RT-PCR (QuantiTect SYBR Green RT-PCR Kit; Qiagen, Hilden, Germany) was conducted to detect DV RNA (using the primer set targeting the NS5 gene) and differentiate DV serotypes (using the four sets of serotype-specific primers targeting the C gene of DENV-1~4) in serum samples from the suspected cases. The serum samples that could not be identified by these four sets of serotype-specific primers were classified as non-differential serotype (NDS).

Patients were conformed as DF or dengue hemorrhagic fever (DHF) by the definition of the 1997 WHO guideline [12]. According to the records of NIDNSS, 6,645 suspected dengue cases were reported to NIDNSS, 3,286 cases were confirmed as DF, and 36 cases confirmed as DHF in Kaohsiung City from 2007 to 2011.

### Collection of epidemiological, clinical and climatic data

The epidemiological and clinical survey of confirmed dengue cases was conducted by local primary health centers in accordance with the Helsinki Declaration. The collected data were then recorded by the Disease Control Office, Department of Health, Kaohsiung City Government (DCO-DH-KCG). The survey included gender, age, residency, travel history, past medical history, manifestations (symptoms and signs), the medical institution for suspected cases to be notified and the time to be notified and confirmed. The recorded data were kept in DCO-DH-KCG for further analysis by academic researchers under permission. This study was approved by the Director of DCO-DH-KCG to use and analyze such data. The data were anonymized and kept confidential. Patients who had not visited a foreign country within the dengue incubation period were classified as “autochthonous” cases; otherwise, they were classified as “imported” cases. The meteorological data on the monthly average temperature, relative humidity, and amount of rainfall were obtained from the Central Weather Bureau of Taiwan.

### Statistical methods

The data were analyzed using the Statistical Package for the Social Sciences for Windows (Version 18.0; IBM Corporation, Armonk, NY, USA). Categorical variables are presented as numbers and percentages. Pearson’s chi-squared test with or without Fisher’s exact test was performed to compare different categorical variables at the two-tailed significance level of 0.05. Fisher’s exact test was used when more than 20% of cells with expected counts of less than 5. The degrees of relationships between dengue cases and various weather parameters (*i*.*e*., temperature, rainfall and relative humidity) both as numerical variables over a range of time lags from 0 to 2 months were determined using correlation and regression analyses.

## Results

### Demographic and epidemiological characteristics of dengue infection

**Fig 1:**
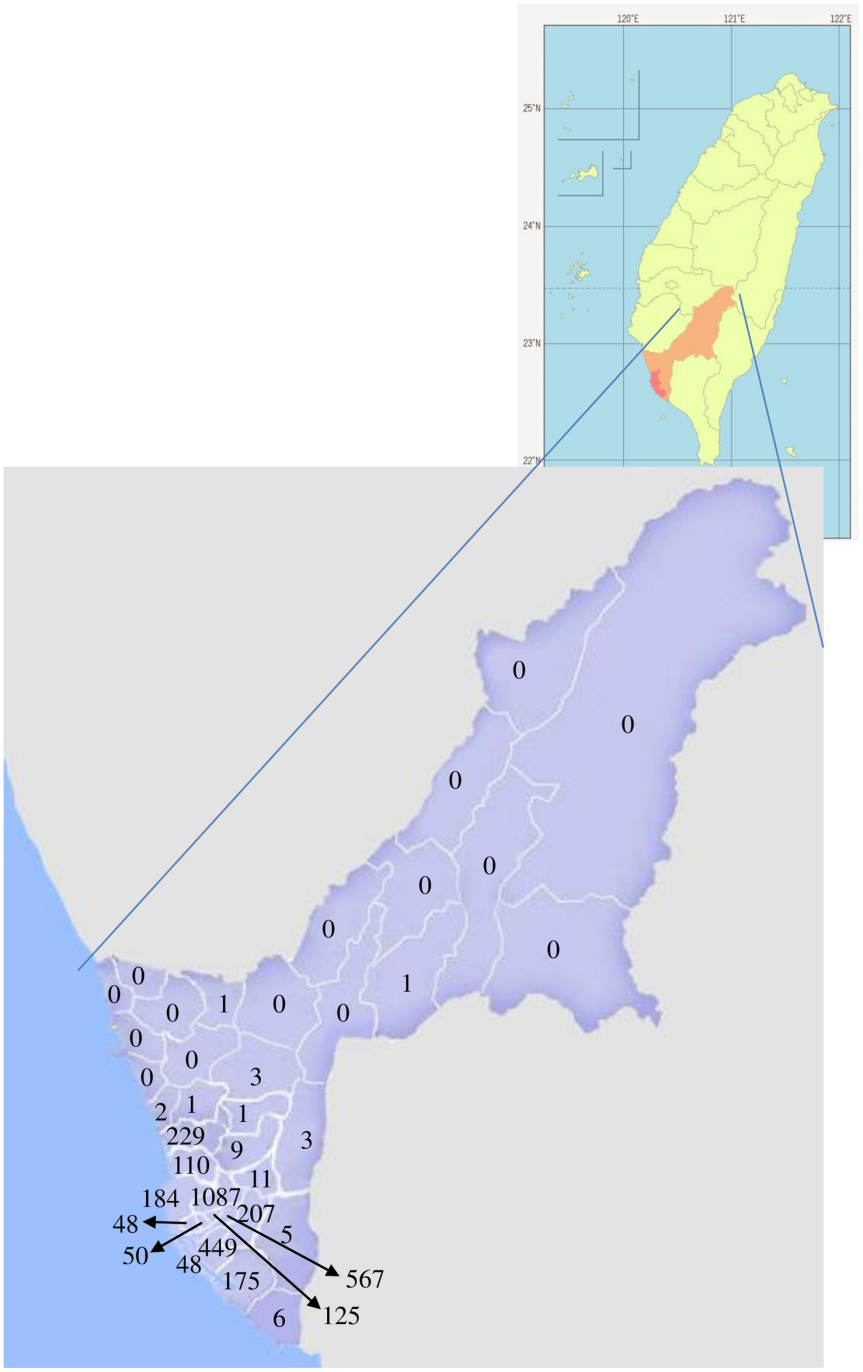
The spatial distribution of dengue patients in Kaohsiung City, Taiwan during 2007–2011.

From 2007 to 2011, a total of 3,322 confirmed dengue cases with an approximately equal distribution of gender each year (*p*> 0.05) were included in the analysis (Table 1). Most (5-year average, 88.2%) confirmed cases were adults (mean age: 42.0, 43.5, 45.5, 43.8, and 45.3 years old for 2007, 2008, 2009, 2010 and 2011, respectively), with the highest percentage shown for the age group of 21–50 years old (Table 1). An average of 70.2% of the patients resided in the early developed urban areas [*i*.*e*., Sanmin District (1087 cases), Linyuan District (567 cases), Cianjhen District (449 cases) and Nanzih District (229 cases)], which have high populations and many man-made containers that serve as larval habitats for dengue mosquitoes (Fig 1). Only a small proportion (5-year average, 2.2%) of confirmed cases was infected in other countries, and of these, most (79.7%) were infected in Vietnam, Indonesia, the Philippines or Thailand (Table 1). DF was observed in 100%, 99.4%, 98.6%, 99.4% and 98.4% of the confirmed cases, while DHF was observed in 0.0%, 0.6%, 1.4%, 0.6% and 1.6% of the confirmed cases in 2007, 2008, 2009, 2010 and 2011, respectively. Death due to DHF occurred in 3, 1 and 4 cases in 2009, 2010 and 2011, respectively (Table 1).

**Table 1:** Demographic and epidemiological characteristics of dengue-infected patients (N = 3,322).

Year	2007	2008	2009	2010	2011	2007–2011
Variables						
**Gender**						
**Female**	66(43.1)	149(43.7)	345(54.2)	551(54.6)	592(50.0)	1,703(51.3)
**Male**	87(56.9)	192(56.3)	291(45.8)	458(45.4)	591(50.0)	1,619(48.7)
**Age groups**						
**0–20**	22(14.4)	53(15.5)	69(10.8)	139(13.8)	142(12.0)	425(12.8)
**21–50**	72(47.0)	151(44.3)	269(42.3)	452(44.8)	533(45.1)	1,477(44.5)
**51–70**	46(30.1)	118(34.6)	256(40.3)	344(34.1)	413(34.9)	1,177(35.4)
**> 70**	13(8.5)	19(5.6)	42(6.6)	74(7.3)	95(8.0)	243(7.3)
**Case source**						
**Autochthonous**	141(92.2)	326(95.6)	623(98.0)	990(98.1)	1,168(98.7)	3,248(97.8)
**Imported**	12(7.8)	15(4.4)	13(2.0)	19(1.9)	15(1.3)	74(2.2)
**Disease type**						
**DF**^a^	153(100.0)	339(99.4)	627(98.6)	1,003(99.4)	1164(98.4)	3,286(98.9)
**DHF**^b^	0(0.0)	2(0.6)	9(1.4)	6(0.6)	19(1.6)	36(1.1)
**Viral serotype**^c^						
**DENV-1**	50(35.5)	138(42.3)	0(0.0)	4(0.4)	2(0.2)	194(6.0)
**DENV-2**	0(0.0)	7(2.2)	1(0.2)	169(17.0)	661(56.6)	838(25.8)
**DENV-3**	0(0.0)	0(0.0)	280(44.9)	322(32.6)	47(4.0)	649(20.0)
**DENV-4**	0(0.0)	1(0.3)	0(0.0)	6(0.6)	0(0.0)	7(0.2)
**NDS**^d^	91(64.5)	180(55.2)	342(54.9)	489(49.4)	458(39.2)	1,560(48.0)
**Infected twice**	1(0.7)	14(4.3)	44(7.1)	54(5.5)	63(5.4)	176(5.4)
**Notification at the first visit**^e^						
**Yes**	18(11.8)	36(10.6)	103(16.2)	260(25.8)	243(20.5)	660(19.9)
**No**	135(88.2)	305(89.4)	533(83.8)	749(74.2)	940(79.5)	2,662(80.1)
**Medical institute to notify dengue cases**						
**Local health center**	51(33.3)	105(30.8)	218(34.3)	237(23.5)	178(15.0)	789(23.8)
**Clinics**	14(9.2)	29(8.5)	31(4.9)	243(24.1)	284(24.0)	601(18.1)
**Community hospital**	3(1.9)	51(15.0)	94(14.8)	80(7.9)	92(7.8)	320(9.6)
**Metropolitan hospital**	35(22.9)	71(20.8)	184(28.9)	215(21.3)	291(24.6)	796(24.0)
**Academic medical center**	45(29.4)	83(24.3)	101(15.9)	230(22.8)	332(28.1)	791(23.8)
**Airport screening**	5(3.3)	2(0.6)	8(1.2)	4(0.4)	6(0.5)	25(0.7)

Data is shown as n(%).

^a^DF: dengue fever.

^b^DHF: dengue hemorrhagic fever.

^c^Virus serotype for autochthonous cases (n = 3,248).

^d^NDS: non-differential serotype

^e^The number of dengue cases was or wasn’t reported to National Infectious Diseases Notification Surveillance System (NIDNSS) at Centers for Disease Control, Taiwan at the first visit to a doctor.

**Fig 2:**
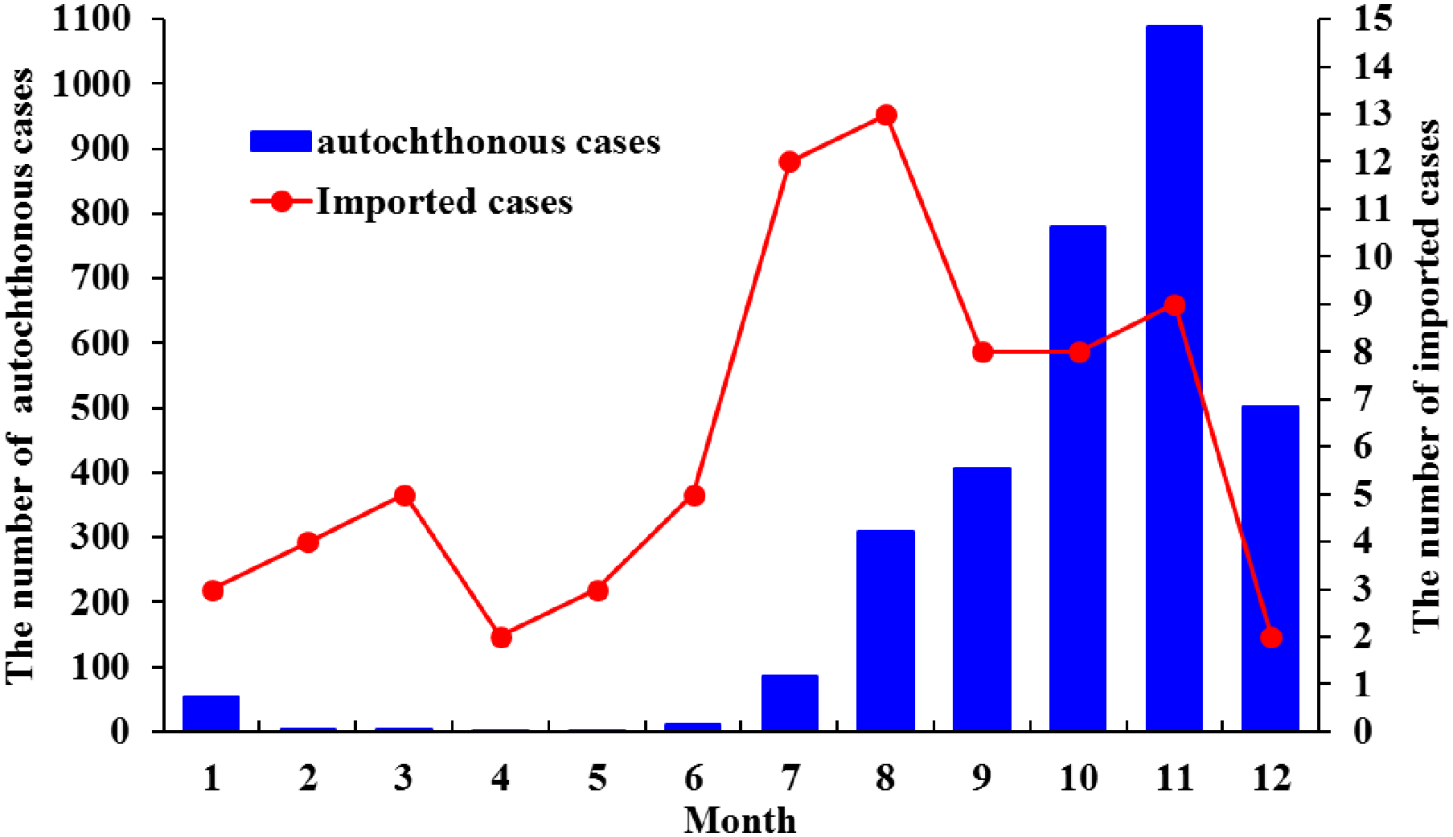
The number of autochthonous and imported dengue patients per month in Kaohsiung City, Taiwan during 2007–2011.

Average 48% of the serum samples from confirmed cases could not be identified as typical DV serotypes (DENV-1~4) and were classified as non-differential serotype (NDS). The distribution of DV serotypes varied throughout the 5-year period. Except for NDS appearing each year, there were 1 (DENV-1), 3 (DENV-1, DENV-2 and DENV-4), 2 (DENV-2 and DENV-3), 4 (DENV-1~4) and 3 (DENV-1~3) serotypes found in 2007, 2008, 2009, 2010 and 2011, respectively (Table 1, Fig 2). Confirmed dengue cases caused by infection with different DV serotypes varied with gender and age (*p*< 0.05, Table 2). Males were more likely to be infected with DENV-1 than females, while female were more likely to be infected with DENV-3 than males (*p*< 0.05, Table 2). The age groups of 0–20 and over 70 years old showed significant difference in infection with DENV-1, while those of 51–70 and over 70 years old showed significant difference in infection with DENV-2 (*p*< 0.05, Table 2). The occurrence of autochthonous dengue cases started from June or July and reached its peak in October or November, then decreased afterward (Fig 2). The imported dengue cases increased from May and reached highest in July and August (Fig 2). The autochthonous and imported patients were mostly infected by DENV-2, with a significant correlation between autochthonous and imported cases in infected serotypes (Spearman’s *ρ* = 0.9, *p* = 0.037). Among the 3,248 autochthonous cases, there were 176 patients infected twice with DV. Most (98.3%) of twice-infected cases belonged to the non-differential serotype at the first infection. At the second infection, 47.2% of twice-infected cases belonged to the non-differential serotype, 30.1% of cases infected with DENV-2, and 19.9% infected with DENV-3. Most (88.6%) of twice-infected cases have fever and nearly half of cases have the symptoms of headache, arthralgia, myalgia, thirsty and loss of appetite. There was no evidence showing that the presence of severe symptoms in the second infection for the twice-infected cases.

**Table 2:** Comparison of four different dengue viral serotypes with gender and age of dengue-infected patients.

		DENV-1 (N = 211)	DENV-2 (N = 857)	DENV-3 (N = 656)	DENV-4 (N = 8)	*p*-value^a^
**Gender**	**Male**	132(62.6)	448(52.3)	292(44.5)	4(50.0)	0.00
	**Female**	79(37.4)	409(47.7)	364(55.5)	4(50.0)	
	*p*-value^b^	< 0.05	> 0.05	< 0.05	> 0.05	
**Age**	**0–20**	33(15.6)	85(9.9)	63(9.6)	0(0.0)	0.02
	**21–50**	100(47.4)	389(45.4)	295(45.0)	3(37.5)	
	**51–70**	69(32.7)	296(34.5)	250(38.1)	5(62.5)	
	**> 70**	9(4.3)	87(10.2)	48(7.3)	0(0.0)	
	***p*-value**^b^	0–20 & >70: < 0.05; the other two groups: > 0.05	51–70 & >70: < 0.05; the other two groups: >0.05	> 0.05	> 0.05	

Data is shown as n(%).

^a^*p*-value: Pearson Chi-square test. The *p*-value represents the differences in frequency of gender or age between the patients infected with 4 different DV serotypes (DENV-1~4).

^b^*p*-value: the post-hoc test using pairwise comparisons with Bonferroni corrections.

Most (5-year average, 80.1%) of the confirmed cases were not reported to NIDNSS at the first visit to a doctor, and most cases were reported at the second visit to a doctor. The major medical institutes to report suspected cases were local health centers, metropolitan hospitals and academic medical centers. The highest number of cases was reported by local health centers from 2007 to 2009 and by clinics and academic medical centers in 2010 and 2011 (Table 1). The least cases were reported by airport screening, accounting for 33.8% (25÷74) of imported cases (Table 1).

### The clinical characteristics of dengue infection

Out of these 3,322 confirmed dengue cases, there were 90.1% that were associated with fever, 51.4% with myalgia, 49.7% with headache and 48.9% with loss of appetite (Table 3). Among the 3,286 patients conformed as DF, most also had fever, myalgia, headache and loss of appetite. Particularly, arthralgia (42.4%) and myalgia (51.6%) were highly correlated with DF (*p*< 0.05). Among the 36 patients conformed as DHF, 83.3% had fever, 52.8% were thirsty, 50.0% had headache, and 44.4% had loss of appetite (Table 3). Most of the cases associated with thirst, loss of appetite, nausea, diarrhea, skin rash and itching were not reported to NIDNSS at the first visit to a doctor (*p*< 0.05) because these symptoms (except for skin rash) were not included in the dengue diagnosis and notification criteria in Taiwan. The confirmed cases showed significantly different clinical manifestations with age (*p*< 0.05), except for hemorrhage and shock (*p*> 0.05, Table 4). There were 795 patients with confirmed cases who had chronic diseases, with hypertension and diabetes being the most common (Table 5). DHF had a significantly stronger association with some chronic diseases, such as hypertension, diabetes, renal diseases, hepatic diseases, cardiovascular diseases, thyroid disease and arthritis, than DF (*p*< 0.05, Table 5).

**Table 3:** Clinical manifestations of dengue-infected patients.

Symptoms	Overall cases (N = 3,322)	DF^a^ (N = 3,286)	DHF^b^ (N = 36)	*p*-value^c^
**Fever**	2,994(90.1)	2,964(90.2)	30(83.3)	0.16*
**Headache**	1,650(49.7)	1,632(49.7)	18(50.0)	0.97
**Retro-orbital pain**	424(12.8)	420(12.8)	4(11.1)	1.00*
**Arthralgia**	1,403(42.2)	1,396(42.4)	7(19.4)	0.01
**Myalgia**	1,707(51.4)	1,696(51.6)	11(30.6)	0.01
**Thirst**	1,251(37.7)	1,232(37.5)	19(52.8)	0.06
**Loss of appetite**	1,623(48.9)	1,607(48.9)	16(44.4)	0.59
**Nausea**	715(21.5)	705(21.5)	10(27.8)	0.36
**Vomiting**	430 (12.9)	422(12.8)	8(22.2)	0.13*
**Diarrhea**	745(22.4)	735(22.4)	10(27.8)	0.44
**Rash**	1,425(42.9)	1,412(43.0)	13(36.1)	0.41
**Itching**	556(16.7)	552(16.8)	4(11.1)	0.36
**Hemorrhage**	113(3.4)	112(3.4)	1(2.8)	1.00*
**Shock**	6(0.2)	6(0.2)	0(0.0)	1.00*

Data is shown as n(%).

^a^DF: dengue fever.

^b^DHF: dengue hemorrhagic fever.

^c^*p*-value: Fisher’s exact test (if more than 20% of cells with expected counts of less than 5) is marked with “*”, while all the other *p* values were calculated by Pearson Chi-square test.

**Table 4:** Comparison of the clinical manifestations with age of dengue-infected patients.

	0–20	21–50	51–70	>70	*p*-value^a^
**Fever**	386(12.9)	812(27.1)	1159(38.7)	637(21.3)	0.00
**Headache**	214(13.0)	475(28.8)	600(36.3)	361(21.9)	0.00
**Retro-orbital pain**	55(13.0)	114(26.9)	160(37.7)	95(22.4)	0.00
**Arthralgia**	180(12.8)	383(27.3)	539(38.4)	301(21.5)	0.00
**Myalgia**	217(12.7)	476(27.9)	660(38.7)	354(20.7)	0.00
**Thirst**	169(13.5)	354(28.3)	486(38.9)	242(19.3)	0.00
**Loss of appetite**	213(13.1)	442(27.2)	646(39.8)	322(19.9)	0.00
**Nausea**	90(12.6)	201(28.1)	286(40.0)	138(19.3)	0.00
**Vomiting**	61(14.2)	118(27.4)	159(37.0)	92(21.4)	0.00
**Diarrhea**	97(13.0)	215(28.9)	304(40.8)	129(17.3)	0.00
**Rash**	193(13.6)	401(28.1)	543(38.1)	288(20.2)	0.00
**Itching**	72(12.9)	167(30.0)	216(38.9)	101(18.2)	0.00
**Hemorrhage**	18(15.9)	35(31.0)	43(38.1)	17(15.0)	0.65
**Shock**	0(0.0)	2(33.3)	1(16.7)	3(50.0)	0.51*

Data is shown as n(%).

^a^*p*-value: Fisher’s exact test (if more than 20% of cells with expected counts of less than 5) is marked with “*”, while all the other *p* values were calculated by Pearson Chi-square test.

**Table 5:** Comparison of chronic diseases in patients with dengue fever and dengue hemorrhagic fever.

Chronic diseases	DF^a^	DHF^b^	*p*-value^c^
**Hypertension**	311(9.5)	15(41.7)	0.00
**Diabetes**	173(5.3)	8(22.2)	0.00
**Stroke**	21(0.6)	1(2.8)	0.21
**Hyperlipidemia**	5(0.2)	1(2.8)	0.06
**Arthritis**	14(0.4)	2(5.6)	0.01
**Asthma**	25(0.8)	0(0.0)	1.00
**Gout**	10(0.3)	0(0.0)	1.00
**Anemia**	7(0.2)	0(0.0)	1.00
**Renal disease**	17(0.5)	3(8.3)	0.00
**Hepatic disease**	68(2.1)	4(11.1)	0.01
**Thyroid disease**	12(0.4)	2(5.6)	0.01
**Stomach disease**	18(0.5)	1(2.8)	0.19
**Cardiovascular disease**	73(2.2)	3(8.3)	0.04

Data is shown as n(%).

^a^DF: dengue fever.

^b^DHF: dengue hemorrhagic fever.

^c^*p*-value: Fisher’s exact test.

The confirmed cases infected with the 4 different DV serotypes showed significant differences in frequency of arthralgia, being thirsty, loss of appetite, nausea, vomiting and diarrhea (Table 6, *p*< 0.05). Among the 4 groups with different DV serotypes, being thirsty and loss of appetite (*p*< 0.05) were significantly more common in the DENV-1 group, while arthralgia (*p*< 0.05) was significantly more common in the DENV-3 group. The patients infected with DENV-3 were more likely to have arthralgia, loss of appetite, nausea, vomiting, and rash than cases with DENV-2 (*p*< 0.05). Patients infected with DENV-2 and DENV-3 tended to be notified at the first or second visit to a doctor (*p*< 0.05), probably because they tended to have conspicuous dengue symptoms, such as fever, headache, arthralgia and myalgia.

**Table 6:** Comparison of the clinical manifestations of dengue-infected patients with four dengue viral serotypes.

	DENV-1 (N = 211)	DENV-2 (N = 857)	DENV-3 (N = 656)	DENV-4 (N = 8)	*p*-value^a^
**Fever**	210(99.5)	826(96.4)	632(96.3)	8(100.0)	0.10
**Headache**	124(58.8)	463(54.0)	372(56.7)	3(37.5)	0.38*
**Retro-orbital pain**	28(13.3)	119(13.9)	96(14.6)	2(25.0)	0.78
**Arthralgia**	96(45.5)	343(40.0)	350(53.4)	4(50.0)	0.00*
**Myalgia**	105(49.8)	497(58.0)	349(53.2)	5(62.5)	0.09*
**Thirst**	111(52.6)	314(36.6)	234(35.7)	2(25.0)	0.00*
**Loss of appetite**	139(65.9)	397(46.3)	344(52.4)	3(37.5)	0.00*
**Nausea**	65(30.8)	155(18.1)	158(24.1)	1(12.5)	0.00
**Vomiting**	41(19.4)	87(10.2)	102(15.5)	1(12.5)	0.00
**Diarrhea**	75(35.5)	158(18.4)	130(19.8)	3(37.5)	0.00
**Rash**	57(27.0)	220(25.7)	203(30.9)	2(25.0)	0.15
**Itching**	24(11.4)	62(7.2)	59(9.0)	0(0.0)	0.17
**Hemorrhage**	11(5.2)	26(3.0)	21(3.2)	0(0.0)	0.42
**Shock**	0(0.0)	4(0.5)	0(0.0)	0(0.0)	0.26*

Data is shown as n(%).

^a^*p*-value: Fisher’s exact test (if more than 20% of cells with expected counts of less than 5) is marked with “*”, while all the other *p* values were calculated by Pearson Chi-square test. The *p*-value represents the difference in frequency of the particular clinical manifestation between the confirmed cases infected with the 4 different DV serotypes.

### Climatic characteristics in relation to dengue occurrence

Most of the confirmed dengue cases occurred at an average temperature of 21–26°C/month, rainfall of ≤ 199 mm/month, and relative humidity of 71–80% (Table 7). Significantly positive correlations (*p*< 0.05) were found between the number of confirmed cases and weather parameters (*i*.*e*., temperature, rainfall and relative humidity) at a time lag of 1 month and 2 months. The regression models that predict occurrence of dengue cases at a time lag of 2 months using temperature, rainfall and relative humidity (individually or together) explained 10%~30% of the variability (shown as R^2^ in Table 7). Although the R^2^ was not high, the models fit the data well with statistical significance (ANOVA F-test, *p*< 0.05, Table 7).

**Table 7:** Climatic characteristics for autochthonous dengue-infected patients (n = 3,248) and statistical analysis between them.

	Cases n(%)	Correlation	Correlation and regression^a^	Correlation and regression^b^
**Average temperature (°C/month)**				
≤ 20	279(8.6)	Spearman’s *ρ* = 0.155,	Spearman’s *ρ* = 0.573, *p* = 0.00*	Spearman’s *ρ* = 0.795, *p* = 0.00*
21–26	1,731(53.3)	*p* = 0.24	Cases = 8.5 T− 160.6,	Cases = 13.6 T− 291.1
≥ 27	1,238(38.1)		R^2^ = 0.1, F = 6.2, *p* = 0.02*	R^2^ = 0.3, F = 19.2, *p* = 0.00*
**Rainfall (mm/month)**				
≤ 199	2,761(85.0)	Spearman’s *ρ* = 0.145,	Spearman’s *ρ* = 0.516, *p* = 0.00*	Spearman’s *ρ* = 0.692, *p* = 0.00*
200–349	39(1.2)	*p* = 0.27	Cases = 0.04 R+ 47.1	Cases = 0.1 R+ 39.0
≥ 350	448(13.8)		R^2^ = 0.02, F = 0.96, *p* = 0.33	R^2^ = 0.1, F = 4.7, *p* = 0.04*
**Relative humidity (%)**				
≤ 70	156(4.8)	Spearman’s *ρ* = 0.336,	Spearman’s *ρ* = 0.625, *p* = 0.00*	Spearman’s *ρ* = 0.754, *p* = 0.00*
71–80	2,864(88.2)	*p* = 0.01*	Cases = 8.0 H− 546.3	Cases = 9.5 H− 652.9
≥ 81	228(7.0)		R^2^ = 0.1, F = 6.3, *p* = 0.02*	R^2^ = 0.1, F = 8.9, *p* = 0.00*
**The regression equation for dengue cases related to three weather parameters**				
			Cases = 5.0 H− 0.1 R+ 7.3 H− 611.7; R^2^ = 0.1, F = 2.7, *p* = 0.06	Cases = 13.9 H+ 0.01 R− 0.9 H− 230.3; R^2^ = 0.3, F = 6.2, *p* = 0.00*

^a^Correlation and regression analysis for the autochthonous dengue cases related to weather parameters at a time lag of 1 month. Nonparametric Spearman correlation was conducted. The goodness of fit of regression models was determined by R^2^ and ANOVA F test.

^b^Correlation and regression analysis for the autochthonous dengue cases related to weather parameters at a time lag of 2 months. Nonparametric Spearman correlation was conducted. The goodness of fit of regression models was determined by R^2^ and ANOVA F test.

T: average temperature; R: rainfall; H: relative humidity.

*: *p*< 0.05

## Discussion

### Identifying risk factors of dengue infection

A significant increasing trend in dengue infection was observed in Kaohsiung City, located in southern Taiwan, as well as in Singapore [13] and Malaysia [14]. The outbreak of DF was mostly found in the early developed urban areas of Kaohsiung. High similarity in the monthly occurrence and DV serotypes of autochthonous and imported cases indicate that DV may have migrated from other countries via imported patients and was transmitted to local hosts, resulting in an epidemic of DF. Most of patients resided in the southern zone near the harbor belonging to the early developed urban areas with high population and many man-made containers. Thus, the rapid spread of dengue in Kaohsiung could be attributed to an increase in urbanization, the use of man-made containers, and international travel/trade. Surveillance for clinical cases (particularly imported cases) and vectors of dengue, therefore, is very important in order to be able to promptly take action to reduce the risk of local transmission in Kaohsiung City, Taiwan.

### Efficiency of the dengue notification system in Kaohsiung City

An effective dengue case notification surveillance system would help to provide the valuable time to make preparations and related control plans. It was found that most of cases were reported by local health centers before 2010. This was because local health centers have a higher awareness of the occurrence of DF than the other medical institutes due to their duty of propagation and investigation of DF. The “Dengue Fever Notification Bonus-penalty Program for Medical Personnel in Kaohsiung” was established and enforced in 2010 to strengthen the awareness about DF by health training and to improve the efficiency of diagnosis and notification. Since then, more cases have been reported by clinics and metropolitan hospitals and academic medical centers. However, fewer cases were reported by community hospitals in 2010–2011 compared to those in 2009, probably because the training for notification of DF were lack in medical workers of community hospitals. One third of imported cases reported by airport screening were due to the appearance of fever, indicating that two third of imported cases were in the incubation period of dengue infection or belonged to asymptomatic dengue infection. In addition, the period for a suspected dengue case being reported from the appearance of the first syndromes decreased from 6 d (median) in 2007 to 4 d (median) in 2011. It indicated that the bonus-penalty policy and health training of medical workers has improved the efficiency of diagnosis and notification of DF.

Many medical workers failed to report suspected dengue cases at their first visit to doctors. It took 5 d (median) after the appearance of the first syndromes for medical personnel to report suspected dengue cases during 2007–2011, while it took 6 d (median) to confirm dengue cases. It was estimated that 95% of the viral incubation period between the time when a mosquito took a viremic bloodmeal and the time when that mosquito became infectious was between 5 and 33 days at 25°C, and 2 and 15 days at 30°C [15]. Thus, *Aedes* mosquitoes would have enough time to be infected with DV by biting individuals infected with DV and transmitting DV before starting quarantine and vector control measures, such as using pesticides and removing water-containing vessels. Such a delay in dengue case reporting has resulted in increasing dengue transmission in Kaohsiung City. It could be attributed to a lack of information regarding the dengue diagnosis guide and dengue notification procedures for medical workers. It was previously determined that complicated dengue notification procedures made medical workers hesitate to notify suspected patients in Taiwan [7]. Thus, it is necessary to simplify dengue notification procedures and educate medical workers about dengue-related knowledge and notification procedures in Taiwan.

### Effects of demographical parameters on dengue infection

The present study and the other four studies conducted in Brazil [16], Singapore [17], India [18] and Saudi Arabia [19] found that dengue mostly affected adults in the age group of 21–70 years old. This may be because adult groups (> 20 years old) engage in more outdoor activities, giving them more chances of being exposed to infected mosquitoes than the younger age group (< 20 years old) [18]. In addition, mild or mainly asymptomatic DV infection was normally found in children [20]. Thus, they were not reported through the active surveillance system.

Previous studies from Cambodia, Malaysia, Sri Lanka, Singapore, the Philippines and India found that males are more prone to DF than females, suggesting that the more common outdoor work habits of males gave them more chances than females to be bitten by mosquitoes [18,21]. However, an equal dengue infection rate was observed in male and female populations in Taiwan in this study, revealing no differences in vector exposure frequency or health care-seeking behavior between males and females. On the other hand, there were gender differences in infection with different DV serotypes that were probably due to different pathogenesis processes or immune responses. More studies are needed to verify this.

### Comparison of clinical manifestations between different studies

Dengue infection has a wide clinical spectrum that varies with different regions and age groups [4,5,22]. Early recognition and understanding of the clinical problems in the febrile phase in a particular region leads to early diagnosis, notification and control of a dengue outbreak. Other than fever, the most frequent symptoms were myalgia, headache, loss of appetite, skin rash, arthralgia and being thirsty in the present study. The notification criteria for suspected cases in Taiwan were the presence of fever and any two of the following symptoms: headache, retro-orbital pain, arthralgia, myalgia, skin rash, hemorrhage and leukopenia. Thus, the loss of appetite and being thirsty should be included in the dengue notification criteria to aid in the early diagnosis of dengue infection. In previous studies, investigators observed that the most frequent symptoms were fever, vomiting, thrombocytopenia and leukopenia in Saudi Arabia [23], while fever, myalgia, arthralgia and headache were the most frequent in Malaysia [5]. It has been suggested that symptoms (e.g., lethargy and thick gallbladder) could be used for the early prediction of severe DHF to avoid its progression to severe dengue [5]; however, in the present study, there were no significant differences in most clinical symptoms between DF and DHF except that arthralgia and myalgia were more closely associated with DF.

This study has shown that the clinical manifestations in dengue patients varied with different DV serotypes, probably due to differences in their pathogeneses, replication ability and infection activity [22]. Skin rash was more commonly found in patients with DENV-3 than DENV-2 in the present and the previous studies [22]; however, arthralgia, loss of appetite, nausea, vomiting were more common in patients with DENV-3 than DENV-2 in the present study, but not in the study by Chan et al. [22]. This may be because the former study investigated all confirmed cases associated with mild and severe symptoms, but the latter study only included patients in hospitals with more severe symptoms. In addition, some chronic diseases, such as hypertension, diabetes, renal diseases, hepatic diseases, cardiovascular diseases, thyroid disease and arthritis, were established to be risk factors for DHF in this study and the previous study by Figueiredo et al.[24], but not that by Chan et al.[22].

### Effects of climatic parameters on dengue infection

It is well known that climate influences dengue ecology by affecting vector dynamics, DV development and mosquito/human interactions, with complicated relationships between climate variables and transmission factors [25]. Temperature was thought to be a key component in dengue viral replication within the vector [26], *Aedes* mosquito population dynamics [27], and the female mosquito’s reproductive cycle [28]. Thus, significant correlations were found between dengue cases/incidence and temperature in the present study and in other studies [9,10,29,30]. It has been found that with every 1°C increase of monthly average temperature, the total population at risk for dengue fever transmission increases by 1.95 times [9]. Temperatures higher than 30°C and lower than 18°C might limit the growth of *Aedes* mosquitoes and DV transmission [10]. Thus, the occurrence of dengue cases sharply decreased from December to January, correlating with the low temperatures (10–18°C) observed in the present study. Rainfall in the man-made containers in urban areas provides habitats for the aquatic stages of the *Aedes* mosquito life cycle. Thus, higher rainfall was associated with increased *Aedes* populations, but intense rainfall might wash out containers and have a negative effect on *Aedes* populations [31]. Accordingly, rainfall was shown to have significant correlations with dengue cases/incidence in some previous studies [30,31], but not for the study in Singapore by Pinto et al. [29]. The present study, in particular, found that dengue cases were significantly positively correlated with rainfall with a time lag of 1 and 2 months, but they did not correlate with rainfall without time lags. Similar conditions were also shown for relative humidity. The reason for this difference may be that relatively higher rainfall and humidity create and maintain breeding sites for *Aedes* mosquitos, and it might take approximately 1 to 2 months for the development of high *Aedes* mosquito populations to transmit DV, resulting in a high occurrence of dengue cases. Brunkard et al. [32] also found lag times for associations between DF incidence and temperature/rainfall in areas along the Texas-Mexico border; however, Wu et al. [9] found a negative association between DF incidence and relative humidity. Therefore, the inconsistent nature of these associations might reflect regional variations in the effects of climate parameters (*i*.*e*., temperature, rainfall and relative humidity) on the occurrence of dengue cases. Thus, based on the 5-year data in the current study, the predictive models for dengue occurrence using temperature, rainfall and relative humidity at a 2-month lag time were derived in Kaohsiung City, which is located in southern Taiwan. These models could be applied to establish an early warning dengue surveillance system for early intervention to reduce the medical, social and economic impact of the disease. Future studies are needed to test and verify these models.

## Conclusion

This study identified imported dengue cases and a delay in reporting suspected cases responsible for the epidemic of dengue fever. The climatic predictive models were first established in Kaohsiung City, Taiwan for early identification of dengue outbreaks. Thus, preventing and controlling dengue outbreaks in Kaohsiung could be through the surveillance of imported cases, adjustment of notification criteria and application of climatic predictive models. The results could be provided to other regions with similar situations for reducing risk of dengue infection.
